# Electrical bioimpedance measurement and near-infrared spectroscopy in pediatric postoperative neurocritical care: a prospective observational study

**DOI:** 10.3389/fneur.2023.1190140

**Published:** 2023-06-21

**Authors:** Chenhao Wang, Dianwei Xing, Shuoyan Zhou, Fang Fang, Yueqiang Fu, Feng Xu

**Affiliations:** ^1^Department of Critical Care Medicine, Children’s Hospital of Chongqing Medical University, Chongqing, China; ^2^National Clinical Research Center for Child Health and Disorders, Chongqing, China; ^3^Ministry of Education Key Laboratory of Child Development and Disorders, Chongqing, China; ^4^Chongqing Key Laboratory of Pediatrics, Chongqing, China

**Keywords:** pediatric brain injury, brain edema, electrical bioimpedance, disturbance coefficient (DC), near-infrared spectroscopy (NIRS), regional cerebral oxygen saturation (rSO_2_)

## Abstract

**Background:**

To investigate the clinical significance of the disturbance coefficient (DC) and regional cerebral oxygen saturation (rSO_2_) as obtained through the use of electrical bioimpedance and near-infrared spectroscopy (NIRS) in pediatric neurocritical care.

**Participants and methods:**

We enrolled 45 pediatric patients as the injury group and 70 healthy children as the control group. DC was derived from impedance analysis of 0.1 mA–50 kHz current via temporal electrodes. rSO_2_ was the percentage of oxyhemoglobin measured from reflected NIR light on the forehead. DC and rSO_2_ were obtained at 6, 12, 24, 48 and 72 h after surgery for the injury group and during the health screening clinic visit for the control group. We compared DC and rSO_2_ between the groups, their changes over time within the injury group and their correlation with intracranial pressure (ICP), cerebral perfusion pressure (CPP), Glasgow coma scale (GCS) score, Glasgow outcome scale (GOS) score, and their ability to diagnose postoperative cerebral edema and predict poor prognosis.

**Results:**

DC and rSO_2_ were significantly lower in the injury group than in the control group. In the injury group, ICP increased over the monitoring period, while DC, CPP and rSO_2_ decreased. DC was negatively correlated with ICP and positively correlated with GCS score and GOS score. Additionally, lower DC values were observed in patients with signs of cerebral edema, with a DC value of 86.5 or below suggesting the presence of brain edema in patients aged 6–16 years. On the other hand, rSO_2_ was positively correlated with CPP, GCS score, and GOS score, with a value of 64.4% or below indicating a poor prognosis. Decreased CPP is an independent risk factor for decreased rSO_2_.

**Conclusion:**

DC and rSO_2_ monitoring based on electrical bioimpedance and near-infrared spectroscopy not only reflect the degree of brain edema and oxygenation, but also reflect the severity of the disease and predict the prognosis of the patients. This approach offers a real-time, bedside, and accurate method for assessing brain function and detecting postoperative cerebral edema and poor prognosis.

## 1. Introduction

Currently, neuroimaging and invasive intracranial pressure (ICP) monitoring are the primary tools utilized for monitoring craniocerebral injury. Neuroimaging methods include computed tomography (CT), magnetic resonance imaging (MRI), transcranial Doppler (TCD), etc. (1). However, these methods are not ideal for clinical monitoring as they are unable to provide real-time, bedside monitoring or reflect dynamic changes in patient’s condition in a timely manner (2). Furthermore, these techniques are associated with high costs and entail a risk of unexpected events during patient transportation. Invasive ICP monitoring methods, including lumbar puncture manometry and intraventricular tube manometry, has technical and invasive procedures, with the potential risk of infections and brain herniation, thereby limiting their clinical applications (3, 4). In recent years, new monitoring technologies based on optical, audiology, ophthalmology, and acoustic principles have been explored, but their clinical value remains to be fully evaluated (5).

The non-invasive cerebral edema dynamic monitor is a non-invasive device based on electrical bioimpedance technology and electromagnetic principles that provide real-time, non-invasive monitoring of intracranial conditions by utilizing disturbance coefficient (DC) as its main output parameter. Previous studies (6, 7) have shown that electrical bioimpedance can be used for the noninvasive assessment of intracranial lesions, thereby avoiding the complications associated with invasive devices. The noninvasive brain oxygenation monitor, which measures regional cerebral oxygen saturation (rSO_2_) through near-infrared spectroscopy (NIRS), is an emerging non-invasive tool for bedside monitoring of brain function. Recent studies (8–13) have suggested that NIRS may help reflect the balance between cerebral perfusion and metabolism in the brain.

Existing research has primarily focused on the clinical application of DC and rSO_2_ in adult patients with brain injury, with limited investigation into their use in the pediatric neurocritical care. This is significant given that brain damage in an immature brain can have far-reaching effects on not just immediate, but also subsequent neurological function. In this study, we aimed to evaluate the clinical significance and potential applications of DC and rSO_2_ in postoperative monitoring of pediatric patients with brain injury, thereby contributing to the field of noninvasive multimodal monitoring in pediatric patients.

## 2. Participants and methods

### 2.1. Participants

This study included 45 pediatric patients who underwent craniocerebral surgery and were admitted to the Department of PICU of a children’s tertiary hospital in western China between March 2021 and October 2022 as the injury group and 70 healthy children seen during the same period as the control group. This study was approved by the Institutional Review Board of the hospital (2021–090) and registered in the Chinese Clinical Trial Registry (ChiCTR2100045057). All participants were fully informed and provided written informed consent for this research. The main inclusion criteria for the injury group were as follows: (i) age between 1 and 16 years; (ii) underwent craniocerebral surgery and admitted to the PICU; and (iii) intact skull without any skin defect near the wing point or above the brow arch. The exclusion criteria were as follows: (i) metal implants in the skull; (ii) dermatosis affecting the electrode stickers; (iii) allergy to the electrode stickers; and (iv) delirium or agitation in patients not receiving analgesic sedation. The main inclusion criteria for the control group were as follows: (i) age between 1 and 16 years; (ii) normal physical and intellectual development; (iii) absence clinical manifestations such as jet vomiting, severe headache, dyspnea, irritability, consciousness impairment, sensory or motor impairments, etc.; (iv) no history of asphyxia, resuscitation, convulsions, encephalitis, meningitis, cranial deformities, trauma, metabolic diseases, serious organ dysfunction, chronic diseases, general anesthesia, no history of cranial hemorrhage, tumor, cyst, hydrocele, edema, vascular malformation, atrophy or related family history; (v) intact skull without any skin defect near the wing point or above the brow arch. The exclusion criteria for the control group were as follows: (i) dermatosis affecting the electrode stickers; (ii) allergy to the electrode stickers; and (iii) inability to cooperate.

### 2.2. Methods

#### 2.2.1. Study protocol

A prospective, observational study was conducted. All patients participating in this study received the same regular medical treatment as the patients who did not participate.

General information (sex, age, weight) and complete medical histories of all participants were recorded. For patients in the injury group, additional date including blood lactate and hemoglobin levels, inspired oxygen concentration (FiO_2_), arterial blood gas analysis (partial pressure of oxygen [PaO_2_], partial pressure of carbon dioxide [PaCO_2_]), the Glasgow Coma Scale (GCS) score before surgery, and the Glasgow Outcome Scale (GOS) score 3 months after surgery were recorded. The results of postoperative head imaging were also documented. For patients who underwent invasive ICP monitoring, ICP and blood pressure obtained by invasive means were also recorded.

For patients in the injury group, DC and rSO_2_ were obtained at 6, 12, 24, 48, 72 h after surgery. DC and rSO_2_ were also monitored continuously from 15 min before to 6 h after the administration of dehydrating drugs. For the healthy children in the control group, DC and rSO_2_ were obtained at the children’s health screening clinic.

#### 2.2.2. Data acquisition

The DC value was obtained using a noninvasive brain edema dynamic monitor (BORN-BE-IV, Chongqing Bornfuke Medical Instrument Co., Ltd., China) based on electrical bioimpedance technology. The monitor comprises two parts: signal acquisition and data processing (Supplementary Figures S1A,B). The monitor can generate a current of less than 2 mA with the working frequency of 10 kHz to 100 kHz. The impedance measurement range is 50 Ω–2000 Ω, with an allowable error of no more than 5%. Four electrode stickers (37 mm × 28.5 mm, Chongqing Bornfuke Medical Instrument Co., Ltd., China) were affixed bilaterally to the temporal region of each participant, with two electrode stickers placed side-by-side on each side (Supplementary Figure S1C). The four electrodes were cycled to act as signal transmitting, signal receiving and grounding electrodes. Prior to electrodes placement, the area was degreased using 75% alcohol. During the measurement, a weak AC current (0.1 mA–50 kHz) was established through one of the electrode stickers, and the differential current was measured by the other two electrode stickers. The DC value was automatically calculated by the monitor by analyzing the amplitude, phase, amplitude slope, and phase slope of the differential current.

The rSO_2_ value was obtained by a noninvasive brain oxygenation monitor (MNIR-P200, Chongqing Bornfuke Medical Instrument Co., Ltd., China) based on NIRS technology (Supplementary Figure S2A). The monitor uses two different wavelengths of NIR light (730 nm and 850 nm) and dual receivers located at 3 cm and 4 cm from the light source to receive the reflected light. The difference in absorption spectra of oxyhemoglobin and deoxyhemoglobin is used to derive regional tissue oxygenation values on the machine, which are displayed as percentages of regional oxyhemoglobin. The probes were placed on the bilateral forehead, with the lower edge about 1 cm from the brow arch and the medial edge at a distance of about 0.5–1 cm from the frontal midline. They were fixed and shaded with disposable non-woven fabric (Supplementary Figure S2B). After placing the probes, immediate figures and trend graphs were available for each side on the monitor.

To determine the DC and rSO_2_ values, the measurement was conducted over a period of 15 min, recorded every 2 s. The average stable value observed during the measurement process was recorded as the measured DC and rSO_2_.

ICP values were obtained by intraventricular monitoring (Codman ICP Express, Johnson & Johnson, United States). Stable ICP values were recorded during monitoring while the patient was in a quiet, supine position.

**Figure 1:**
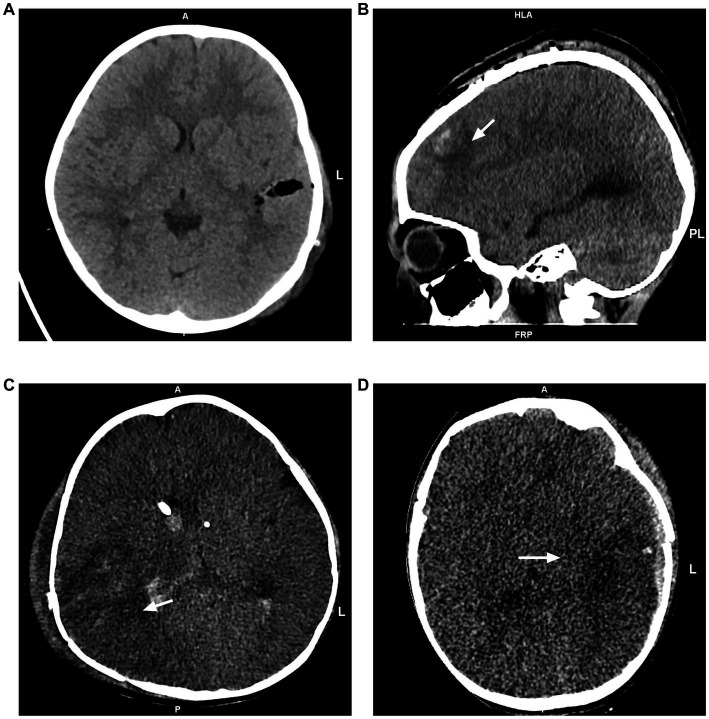
Postoperative head CT images. **(A)** No edema was observed. **(B)** Focal edema limited to the left frontal lobe. **(C)** Unilateral edema in the right temporal, occipital, and parietal lobes. **(D)** Global edema in the left cerebral hemisphere.

Postoperative head CT examinations were performed using a CT scanner with 16-slice scanner (Somatom Emotion, Siemens Company, Germany) or 64-slice scanner (Light Speed VCT, GE Medical System, United States). Referring to Lietke et al. (14), postoperative head CT findings were categorized as no edema, focal edema (edema limited to one lobe) and multifocal edema (unilateral edema affecting more than one lobe, bilateral edema or global edema; Figure 1). Mean arterial pressure (MAP) was calculated from radial artery invasive blood pressure monitoring data by the formula:


$$\mathrm{MAP}=\left(\mathrm{systolic pressure}+2\times \mathrm{diastolic pressure}\right)÷3.$$

Cerebral perfusion pressure (CPP) was computed as: CPP=MAP–ICP.

**Figure 2:**
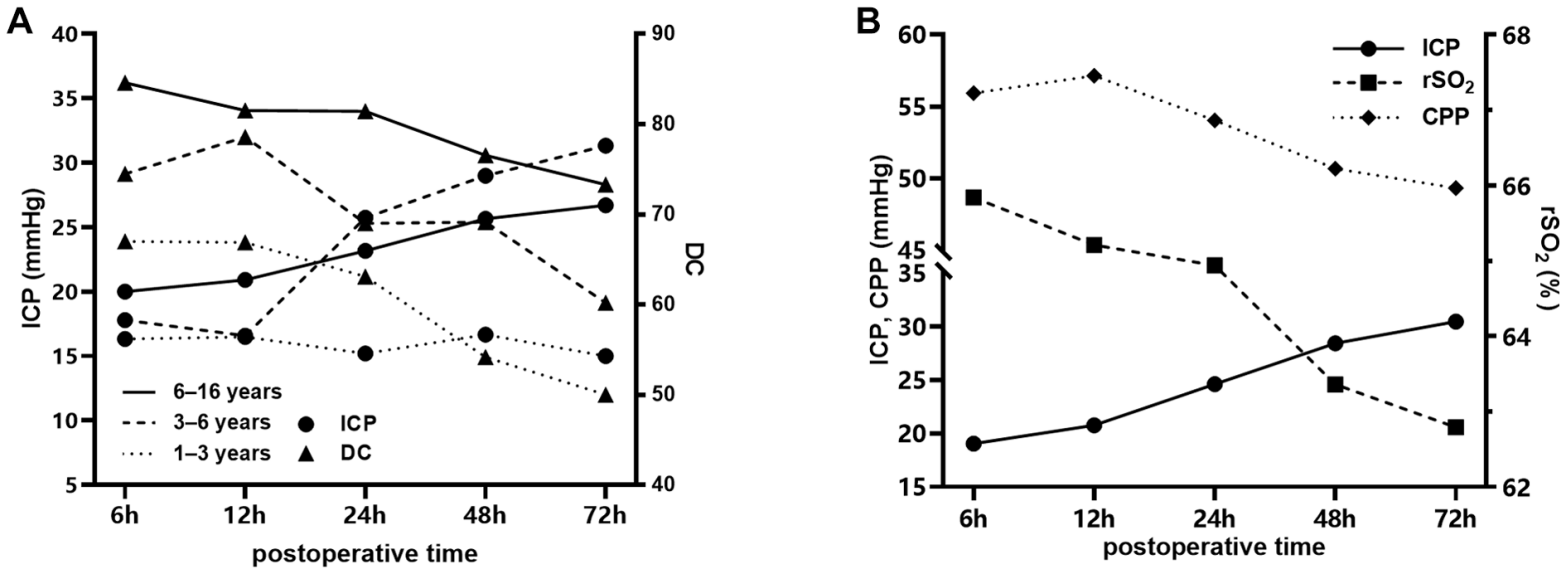
Temporal changes in different parameters after craniocerebral surgery in the injury group. **(A)** Changes in DC and ICP in patients aged 1–3 years (dotted line), 3–6 years (dashed line) and 6–16 years (solid line) after craniocerebral surgery. **(B)** Changes in rSO_2_, ICP and CPP in patients aged 1–16 years after craniocerebral surgery.

### 2.3. Statistical analysis

The Case Record Form (CRF) was used for data collection. SPSS (version 24, SPSS Inc., Chicago, IL, United States) and R software (Version 4.2.1, R Foundation for Statistical Computing, Vienna, Austria) were used for data processing. Normality tests were performed using the Shapiro–Wilk test. The measurement data conforming to the normal distribution are expressed as the mean ± standard deviation (SD), *t* tests were used for comparisons between two groups, and ANOVA was used for comparisons between multiple groups. Measurement data that did not conform to a normal distribution were expressed as the median (*Q*1, *Q*3), and the rank-sum test was used for comparisons between groups. Enumeration data are expressed as cases and percentages (*n*, %), and the chi-square test was used for comparisons between groups. Scatter plots and linear correlation analysis were used to analyze the correlations between parameters. Diagnostic tests were evaluated by receiver operating characteristic (ROC) curves. The area under the ROC curve (AUC), 95% confidence interval (*CI*), and corresponding sensitivity and specificity were also calculated. Time series data were analyzed with a generalized linear model using a normal distribution and identity-link function and were extended by generalized estimating equations (GEEs) with an autoregressive process of the first order to handle repeated observations within a subject (15). Repeated measures correlation was performed by a repeated measures correlation analysis using an R software package named “rmcorr” (16). *p* < 0.05 was considered statistically significant.

## 3. Results

### 3.1. Participant characteristics

A total of 70 healthy children and 45 pediatric patients were studied, including 35 males and 35 females in the control group and 24 males and 21 females in the injury group. The median age of children in the control group was 5.3 (2.8–9.3) years, and the median age of children in the injury group was 6.2 (3.1–9.7) years. In the injury group, the median GCS score was 8.5 (5.8–15.0) and the median GOS score was 3.5 (2.0–5.0); 4 children (8.9%) died, 25 (55.6%) received invasive ICP monitoring, 35 (77.8%) received mechanical ventilation; the median duration of mechanical ventilation was 1.0 (0.5–4.3) days, the median length of stay was 24.0 (11.5–39.5) days, and the median PICU length of stay was 3.5 (2.0–9.0) days.

### 3.2. Comparison of DC and rSO_2_ between the injury and control groups

Tables 1, 2 demonstrate the differences in DC and rSO_2_ between the injury and control group participants of different age groups. The results show that DC and rSO_2_ were significantly lower in the injury group in all age groups.

**Table 1:** Comparison of DC in patients of different ages in the control and injury groups.

Age	Control group	Injury group	*t* value	*p* value
>1 and ≤ 3 yr	76.95 ± 9.80	66.15 ± 14.37	−2.48	0.019
>3 and ≤ 6 yr	83.65 ± 11.84	71.73 ± 15.58	−2.40	0.023
>6 and ≤ 16 yr	98.47 ± 7.94	84.37 ± 14.67	−4.48	<0.001

DC, disturbance coefficient.

Data are presented as the mean ± standard deviation. The sample size for each age group was as follows: control group: *n* = 20 (>1 and ≤ 3 yr), *n* = 20 (>3 and ≤ 6 yr), *n* = 30 (>6 and ≤ 16 yr); injury group: *n* = 11 (>1 and ≤ 3 yr), *n* = 11 (>3 and ≤ 6 yr), *n* = 23 (>6 and ≤ 16 yr).

**Table 2:** Comparison of rSO_2_ in patients of different ages in the control and injury groups.

Age	Control group	Injury group	*Z* value	*p* value
>1 and ≤ 3 yr	67.0 (66.0–68.0) (%)	66.3 (64.1–66.9) (%)	−2.08	0.038
>3 and ≤ 6 yr	67.5 (67.0–68.5) (%)	66.4 (63.0–67.0) (%)	−2.44	0.015
>6 and ≤ 16 yr	68.3 (67.0–69.5) (%)	66.8 (65.1–68.0) (%)	−2.97	0.003

rSO_2_, regional cerebral oxygen saturation.

Data are presented as the median (Q1, Q3). The sample size for each age group was as follows: control group: *n* = 20 (>1 and ≤ 3 yr), *n* = 20 (>3 and ≤ 6 yr), *n* = 30 (>6 and ≤ 16 yr); injury group: *n* = 10 (>1 and ≤ 3 yr), *n* = 10 (>3 and ≤ 6 yr), *n* = 23 (>6 and ≤ 16 yr).

### 3.3. Temporal changes in DC and rSO_2_ in the injury group

Figure 2 shows the trend of DC, rSO_2_, ICP, and CPP values over time in patients in the injury group at 6–72 h after surgery. Figure 2A demonstrate the temporal changes of DC and ICP in injury group of different age. A decreasing trend of DC was observed in all age groups, and ICP showed an increasing trend (*p* < 0.001) in all age groups except for the 1–3-year-old group where the increasing trend of ICP was not statistically significant (*p* = 0.056). Figure 2B shows the temporal changes of rSO_2_, CPP, and ICP of all ages, with a decreasing trend of rSO_2_ and CPP and an increasing trend of ICP, all of which were statistically significant (*p* < 0.001). DC and rSO_2_ remained stable during dehydration drug use (30 min before to 6 h after).

### 3.4. Comparison of DC and rSO_2_ in patients with different characteristics in the injury group

Among the patients in the injury group, statistically significant differences in DC among patients of different ages were found, while the differences in rSO_2_ were not statistically significant.

**Figure 3:**
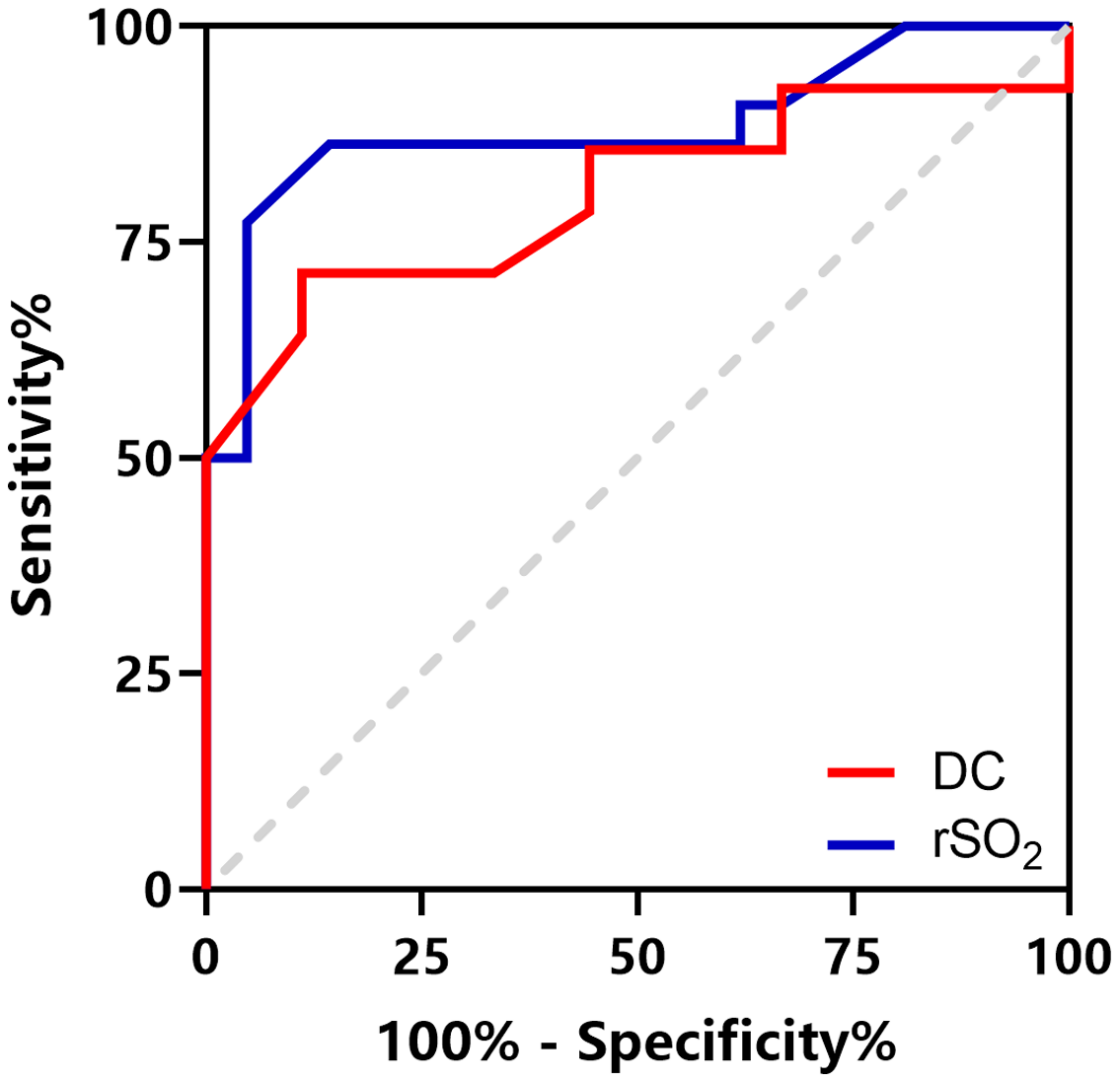
ROC curves for DC to identify brain edema in patients aged 6–16 years (red line) and rSO_2_ to predict a poor prognosis in patients aged 1–16 years (blue line).

DC was significantly lower in patients with CT evidence of brain edema (Table 3). Among the 14 patients aged 6–16 years with signs of brain edema, 7 were identified with focal edema and 7 with multifocal edema. Patients with multifocal edema had lower DC values than those with focal edema (71.57 ± 12.95 vs. 87.71 ± 12.18, *p* = 0.033). ROC curve analysis revealed that DC had excellent diagnostic ability for brain edema in patients aged 6–16 years old with an AUC value of 0.806 (*95% CI* 0.624–0.987, *p* = 0.015, Figure 3). The optimal cutoff value of DC was 86.5, with a sensitivity of 71.4% and specificity of 88.9%.

**Table 3:** Comparison of DC and rSO_2_ in patients with different characteristics in the injury group.

Characteristics		*N*	DC	*p* value	rSO_2_ (%)	*p* value
Gender	Male	24 (53.3)	77.33 ± 14.18	*p* > 0.05	66.5 (64.8–67.3)	*p* > 0.05
	Female	21 (46.7)	76.25 ± 19.29		66.2 (65.2–67.3)	
Causes	Hematoma	27 (60)	76.28 ± 16.96	*p* > 0.05	66.0 (64.3–66.9)	*p* > 0.05
	Brain contusion	6 (13.3)	68.40 ± 10.98		66.8 (63.2–67.4)	
	Tumor	10 (22.2)	78.80 ± 16.30		67.2 (66.2–68.1)	
	Hydrocephalus	2 (4.4)	99.60 ± 9.76		67.7 (−)	
Surgery	Hematoma removal	23 (51.1)	74.77 ± 17.42	*p* > 0.05	65.4 (64.1–66.6)	*p* > 0.05
	Tumor resection	10 (22.2)	78.80 ± 16.30		67.2 (66.2–68.1)	
	Brain contusion debridement	6 (13.3)	68.40 ± 10.98		66.8 (63.2–67.4)	
	Ventricle drainage	6 (13.3)	89.87 ± 12.85		67.4 (66.8–70.8)	
Head CT	Brain edema (+)	25 (55.6)	69.28 ± 17.51	*p* = 0.001	64.8 (61.8–67.2)	*p* > 0.05
	Brain edema (−)	20 (44.4)	85.20 ± 12.18		66.5 (66.0–67.9)	
GCS score	≤8	19 (42.2)	68.01 ± 14.35	*p* = 0.001	65.1 (61.1–66.9)	*p* = 0.007
	≥9	26 (57.8)	83.28 ± 15.25		66.9 (66.2–67.9)	
GOS score	≤3	22 (48.9)	66.84 ± 13.36	*p* < 0.001	65.2 (61.6–67.0)	*p* = 0.001
	≥4	23 (51.1)	86.39 ± 13.54		66.9 (66.3–68.0)	

DC, disturbance coefficient; rSO_2_, regional cerebral oxygen saturation; CT, computed tomography; GCS, Glasgow Coma Scale; GOS, Glasgow Outcome Scale.

Data are presented as n (%), mean ± standard deviation or median (Q1, Q3).

DC and rSO_2_ in the severe group (GCS score ≤ 8) were significantly lower than those in the mild-to-moderate group (GCS score ≥ 9), and the differences were statistically significant (Table 3). Correlation analysis suggested that DC and rSO_2_ were positively correlated with GCS score in the injury group (*r_DC_* = 0.568, *p* < 0.001; *r_rSO2_* = 0.550, *p* < 0.001).

DC and rSO_2_ values in patients with poor prognosis (GOS score ≤ 3) were significantly lower than those in the favorable prognosis group (GOS score ≥ 4; Table 3). Correlation analysis suggested that DC and rSO_2_ were positively correlated with GOS (*r_DC_* = 0.685, *p* < 0.001; *r_rSO2_* = 0.701, *p* < 0.001). ROC curve analysis suggests that a minimum rSO_2_ < 64.4% can be regarded as an indicator for predicting poor prognosis with a sensitivity of 77.3% and a specificity of 95.2%. The AUC was 0.883 (*95% CI* 0.774–0.992, *p* < 0.001, Figure 3).

**Figure 4:**
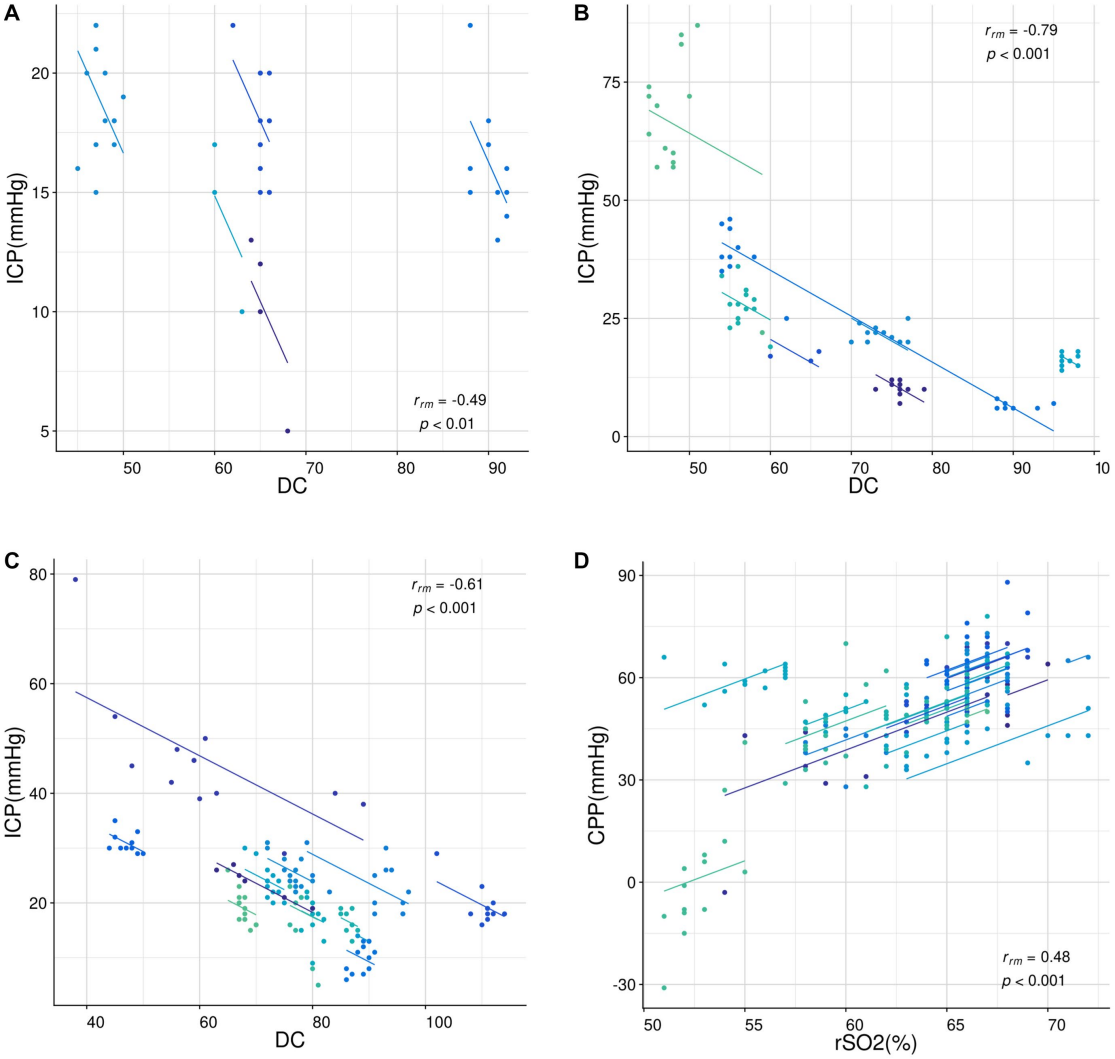
Correlation analysis of repeated measurement data. **(A-C)** The relationship between DC and ICP in patients aged 1–3 years **(A)**, 3–6  years **(B)**, and 6–16 years **(C)**. **(D)** The relationship between rSO_2_ and CPP in patients aged 1–16 years. All data were analyzed using the “rmcorr” package in R language. The dots represent the data points and the lines represent the linear regression lines. The correlation coefficients (*r_rm_*) and *p*-values are shown in each sub-figure.

Correlation analysis of repeated measurement data suggests a negative correlation between DC and ICP in patients of different age groups (*r_A_* = −0.49, *p* = 0.002; *r_B_* = −0.79, *p* < 0.001; *r_C_* = −0.61, *p* < 0.001, Figures 4A–C) and a positive correlation between rSO_2_ and CPP in patients of all ages (*r_D_* = 0.48, *p* < 0.001, Figure 4D).

There were no statistically significant differences in DC and rSO_2_ between different sexes, causes, and types of surgery. No correlation was found between DC, rSO_2_ and PICU length of stay, hospital length of stay, duration of mechanical ventilation and hemoglobin and lactate levels at the corresponding time points.

### 3.5. Risk factors for postoperative cerebral desaturation

Patients who experienced ICP ≥20 mmHg, CPP ≤55 mmHg and PaO_2_/FiO_2_ < 300 had a 3.29-fold, 8.73-fold, and 2.60-fold increased risk of cerebral desaturation (defined as rSO_2_ < 65%), respectively (Table 4). In the severe group, patients who experienced severe hypocapnia (PaCO_2_ < 30 mmHg) had a 5.56-fold increased risk of decreased cerebral oxygen saturation (Table 5). Multivariate analysis suggested that CPP ≤55 mmHg was associated with desaturation (Table 4), while in the severe group, the risk of desaturation was elevated 10.91-fold (Table 5).

**Table 4:** Risk factors for postoperative desaturation in injury group.

Characteristic	Univariate analysis		Multivariate analysis	
	RR (95% *CI*)	*p* value	RR (95% *CI*)	*p* value
CPP				
>55 mmHg	1^*^	–	1^*^	–
≤55 mmHg	9.73 (2.94–32.22)	<0.001	6.01 (1.78–20.27)	0.004
ICP				
<20 mmHg	1^*^	–	1^*^	–
≥20 mmHg	4.29 (1.33–13.85)	0.015	2.46 (0.60–10.10)	0.213
PaO_2_/FiO_2_				
≥300	1^*^	–	1^*^	–
<300	3.60 (1.26–10.25)	0.016	1.74 (0.53–5.75)	0.303
PaCO_2_				
≥35 mmHg	1^*^	–	1^*^	–
30-35 mmHg	1.11 (0.42–2.95)	0.829	1.44 (0.32–6.52)	0.635
<30 mmHg	2.10 (0.67–6.55)	0.203	0.45 (0.18–1.13)	0.091

RR, risk ratio; CI, confidence interval; CPP, cerebral perfusion pressure; ICP, intracranial pressure; PaO_2_, partial pressure of oxygen; FiO_2_, fraction of inspired oxygen; PaCO_2_, partial pressure of carbon dioxide.

^*^ Reference category.

**Table 5:** Risk factors for postoperative desaturation in severe craniocerebral injury subgroup.

Characteristic	Univariate analysis		Multivariate analysis	
	RR (95% *CI*)	*p* value	RR (95% *CI*)	*p* value
CPP				
>55 mmHg	1^*^	–	1^*^	–
≤55 mmHg	6.96 (1.63–29.63)	0.009	10.91 (2.57–46.29)	0.001
ICP				
<20 mmHg	1^*^	–	1^*^	–
≥20 mmHg	1.24 (0.43–3.61)	0.693	0.89 (0.16–4.82)	0.888
PaO_2_/FiO_2_				
≥300	1^*^	–	1^*^	–
<300	3.05 (0.65–14.32)	0.158	1.40 (0.43–4.49)	0.577
PaCO_2_				
≥35 mmHg	1^*^	–	1^*^	–
30-35 mmHg	1.09 (0.30–4.02)	0.893	0.61 (0.20–1.89)	0.391
<30 mmHg	6.56 (1.19–36.31)	0.031	1.78 (0.29–10.94)	0.535

RR, risk ratio; CI, confidence interval; CPP, cerebral perfusion pressure; ICP, intracranial pressure; PaO_2_, partial pressure of oxygen; FiO_2_, fraction of inspired oxygen; PaCO_2_, partial pressure of carbon dioxide.

^*^ Reference category.

## 4. Discussion

Secondary damage occurs several hours and days after injury due to complex mechanisms, including free-radical generation, depolarization, disruption of the blood–brain barrier, and calcium homeostasis triggered by primary damage (17). These cascades lead to cerebral swelling, brain edema and increased ICP. Intracranial hypertension impairs CPP and cerebral blood flow (CBF) according to the Monro-Kellie theory, thereby causing cerebral hypoxia and aggravating cerebral edema (18). Our study demonstrated temporal changes in ICP and CPP in pediatric patients after craniocerebral surgery, which is in line with current studies and clinical observations (19, 20). The changes in DC and rSO_2_ are also consistent with these findings, suggesting that DC and rSO_2_ could reflect the postoperative pathophysiological changes in pediatric patients.

Lower DC value was associated with higher ICP and might help diagnose postoperative brain edema. Electrical bioimpedance reflects the electrical characteristics of biological cells, tissues or organs (21), and DC is a customized parameter based on this technology. It is a numerical representation of the electrical properties of biological tissues. Previous study has shown that edema events can lead to a decrease DC, and a decrease in DC value greater than 18 indicates hydrocephalus (22). The conductivity and permittivity of intracranial tissues are altered in postoperative patients due to vasogenic and cytotoxic edema (23, 24), which in turn leads to lower DC in postoperative children compared to healthy children. He et al. (25) found that perturbative index (PI), based on electrical bioimpedance technology, correlated with the infarction side and volume of infarction (r = 0.682) in patients with cerebral infarction. A study by Fu et al. (26) found that impedance values increased (from 0.13 ± 0.07 to 0.82 ± 0.15) and ICP decreased (from 17.9 ± 5.1 to 12.1 ± 1.7 mmH_2_O) during the use of dehydrating drugs in patients with cerebral hemorrhage. We observed a negative correlation between DC and ICP in pediatric patients who underwent craniocerebral surgery. This reflects the applicability of DC monitoring to pediatric patients. Furthermore, our study found that patients with signs of brain edema suggested by postoperative head CT had significantly lower values of DC, with more severe edema being associated with lower DC values. According to our ROC curve analysis, DC exhibited excellent ability in detecting brain edema. In pediatric patients aged 6 years and older, DC ≤86.5 may indicate the existence of brain edema. Therefore, as a noninvasive and bedside monitoring tool, DC monitoring shows the potential to identify postoperative intracranial hypertension and cerebral edema in children.

We also found a significant decrease in rSO_2_ and a positive correlation between rSO_2_ and CPP in pediatric patients who underwent craniocerebral surgery. An increasing number of studies (27, 28) suggest that NIRS, as a noninvasive method, allows rapid, continuous and measurable detection of brain oxygenation, and the information regarding the balance between oxygen supply and oxygen consumption can be analyzed through rSO_2_ (29). Secondary brain injury in postoperative craniocerebral injury patients leads to intracranial hypertension and impaired cerebral blood flow autoregulation, causing a decrease in cerebral blood flow and resulting in lower rSO_2_. These findings follow earlier studies (30, 31) in adult patients with traumatic brain injury (TBI). CPP represents the pressure gradient that drives CBF. Sufficient CPP can ensure an oxygen supply to brain tissue and prevent ischemia and hypoxia. The moderate correlation between rSO_2_ and CPP suggests that rSO_2_ monitoring has potential value in pediatric neurocritical care.

Risk factor analysis suggests that desaturation in pediatric patients who underwent craniocerebral surgery may be associated with severe hypocapnia, impaired systemic oxygenation and decreased CPP. Results showed that severe hypocapnia (PaCO_2_ < 30 mmHg) may increase the risk of cerebral ischemia in severely injured patients, but mild hypocapnia (30 mmHg ≤ PaCO_2_ < 35 mmHg) appears safer. This finding is consistent with the findings of Adelson et al. (32) in a preliminary study, who found that decreased PaCO_2_ was associated with cerebral oxygen desaturation in pediatric patients. Hyperventilation is now deemed the second-tier therapy in patients with increased ICP (33) through hypocapnia-induced cerebral vasoconstriction. However, it produces hypoperfusion or ischemia, and concerns have been raised about its safety (34). Our study also found that lower PaO_2_/FiO_2_ may increase the risk of desaturation in the severe group, which indicates that systemic oxygenation also appears to correlate with cerebral oxygenation in pediatric patients. This finding is in accordance with reports from Robba et al. (35), who analyzed brain and systemic oxygenation and concluded that PaO_2_/FiO_2_ was a strong and independent risk factor for cerebral hypoxia and an independent predictor of mortality in adult patients with TBI. Therefore, maintaining appropriate systemic oxygenation seems to be critical for pediatric patients with brain injury. Multivariate analysis suggested that patients who experienced CPP ≤55 mmHg had a higher risk of experiencing cerebral oxygen desaturation, which is in substantial agreement with Davie et al. (36), who found that adult patients with TBI with elevated ICP were 6 times more likely to have desaturation. The treatment threshold of CPP may be age dependent, and guidelines (37) suggest a CPP target between 40 mmHg and 50 mmHg. However, current research (38) suggests that this target may not be sufficient, especially for children over 2 years of age. Chen et al. (39) reported that the optimal treatment threshold for CPP in children aged 2 years older might be 55 mmHg. Our study supported this idea in another way.

Lower DC and rSO_2_ levels were associated with more severe disease and poor prognosis. The GCS is commonly used to determine the severity of neurological disease (40). However, accurate assessment of the neurological status of pediatric patients with developmental delays or those undergoing sedation or mechanical ventilation can be challenging, relying heavily on experience and training (41). Therefore, these bedside monitoring tools might serve as a useful supplement to GCS for assisting in the assessment of patient’s condition. The GOS is the most widely used tool to assess the prognosis of brain injury (42). ROC curve analysis showed that rSO_2_ had excellent prognostic predictive ability, and rSO_2_ ≤ 64.4% indicated poor prognosis. Vilkė et al. (43) found that rSO_2_ < 68% at 1 h after admission to the ICU was associated with an increased risk of death. The numerical differences may be related to the different ages of patients in the included populations. In another study by Durnev et al. (30), a possible association was found between elevated rSO_2_ levels relative to baseline and poor outcomes in terms of neurological sequelae. Since rSO_2_ reflects the balance between oxygen supply and oxygen consumption, a decrease in rSO_2_ suggests a reduction in blood supply. Conversely, increased rSO_2_ may indicate either a decrease in the metabolic demand of brain tissue or the existence of regional ischemia.

Undeniably, we acknowledge that this study has several limitations. Firstly, the sample size of the study was relatively small, especially in the younger age groups. Secondly, the water content of the brain varies among children of different ages, which affects the normal value of DC. Previous studies have found that DC values become stable after the age of 5 to 6 years and the wide age interval of our study population may have impacted the results.

In this observational study, we monitored DC and rSO_2_ using electrical bioimpedance and NIRS techniques in pediatric patients after craniocerebral surgery, and explored their clinical significance. Results showed that DC and rSO_2_ were significantly different between the injury and control groups, and were found to decrease with the increase of ICP and increase with the increase of CPP in the injury group. We also found that DC could be used to diagnose postoperative cerebral edema, and rSO_2_ could be used to predict poor prognosis. The monitoring of DC and rSO_2_ provides a real-time, bedside, and accurate method for assessing brain function in pediatric neurocritical care, which might help guide clinical treatment and intervention. Further studies should investigate interventions to determine whether early interventions for DC and rSO_2_ improve the prognosis of children.

## Data availability statement

The raw data supporting the conclusions of this article will be made available by the authors, without undue reservation.

## Ethics statement

The studies involving human participants were reviewed and approved by Institutional Review Board of Children’s Hospital of Chongqing Medical University. Written informed consent to participate in this study was provided by the participants' legal guardian/next of kin.

## Author contributions

FX, FF, YF, and CW contributed to conception and design of the study. CW, DX, SZ, and YF contributed to data acquisition. CW, DX, SZ, FF, YF, and FX organized and analyzed the data. CW wrote the manuscript. FF and FX revised the manuscript. All the authors contributed to the article and approved the submitted version.

## Funding

This research was funded by the Key Project of the Technology Innovation and Application Development Program of Chongqing (cstc2020jscx-sbqw0006), the Project of the National Clinical Research Center for Child Health and Disorders of China (NCRCCHD-2020-GP-08) and the Natural Science Foundation Project of Chongqing of China (cstc2020jcyj-msxmX1087).

## Conflict of interest

The authors declare that the research was conducted in the absence of any commercial or financial relationships that could be construed as a potential conflict of interest.

## Publisher’s note

All claims expressed in this article are solely those of the authors and do not necessarily represent those of their affiliated organizations, or those of the publisher, the editors and the reviewers. Any product that may be evaluated in this article, or claim that may be made by its manufacturer, is not guaranteed or endorsed by the publisher.
