# Supplementary material for: Electrical bioimpedance measurement and near-infrared spectroscopy in pediatric postoperative neurocritical care: a prospective observational study
Source: Front Neurol. 2023 Jun 21;14:1190140. doi: 10.3389/fneur.2023.1190140 (PMC10322191; doi:10.3389/fneur.2023.1190140)

Supplementary Material

Electrical Bioimpedance Measurement and Near-Infrared Spectroscopy in Pediatric Postoperative Neurocritical Care: A Prospective Observational Study

Chenhao Wang^1^, Dianwei Xing^1^, Shuoyan Zhou^1^, Fang Fang^1*^, Yueqiang Fu^1^, Feng Xu^1*^

*** Correspondence:**

Feng Xu, xufeng9899@163.com

Fang Fang, fangfangviking@sina.com

Supplementary Figure 1. Noninvasive brain edema dynamic monitor based on electrical bioimpedance technology. (**A**) The setup of the device for monitoring pediatric patients after craniocerebral surgery in the pediatric intensive care unit. (**B**) Hardware setup of the device. (**C**) Placement of electrode stickers on a patient's left temporal region (same for both sides).


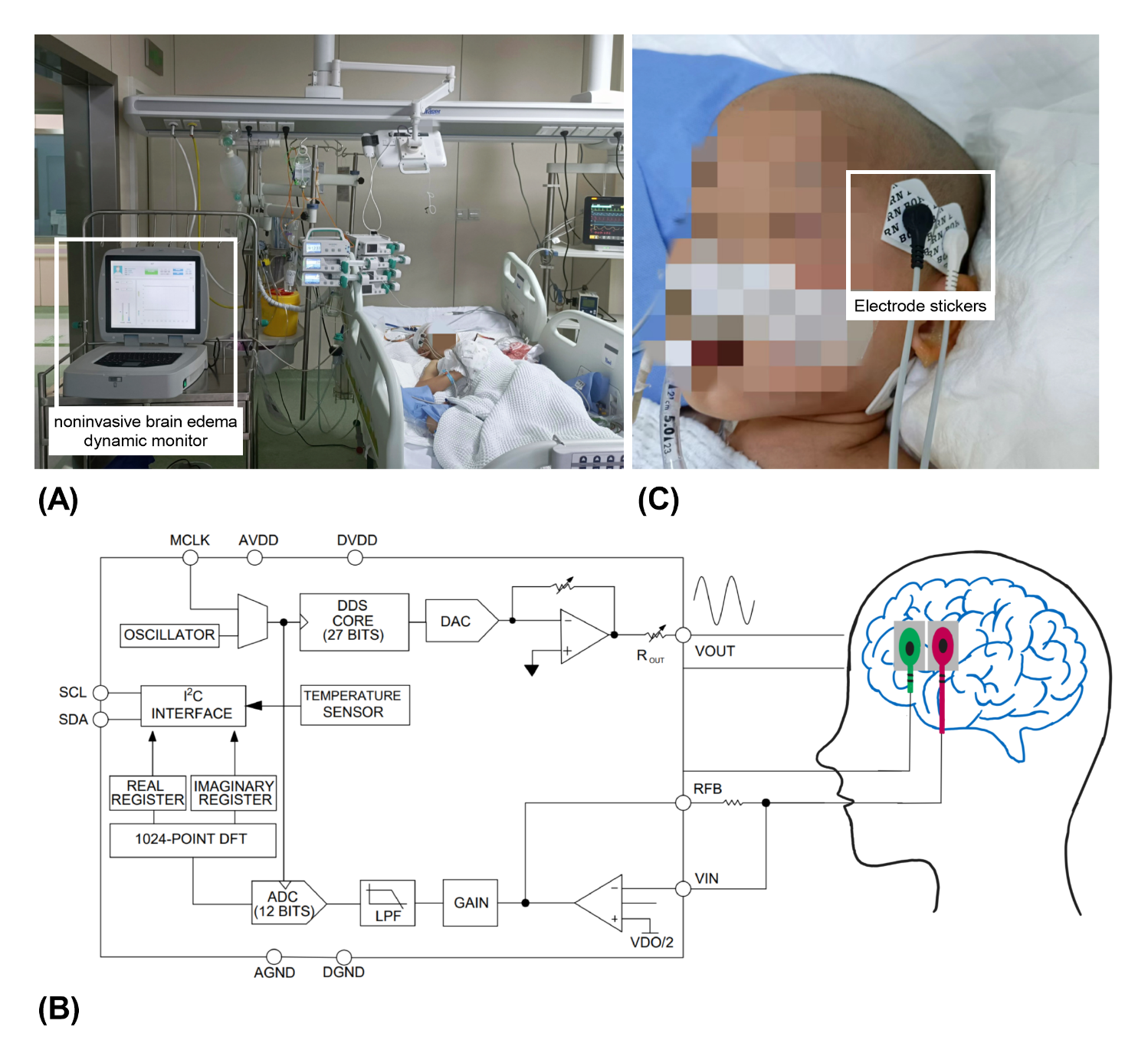


Supplementary Figure 2. Noninvasive brain oxygenation monitor based on near-infrared spectroscopy. (**A**) The setup of the device for monitoring pediatric patients after craniocerebral surgery in the pediatric intensive care unit. (**B**) Probes placed on a patient's bilateral forehead.


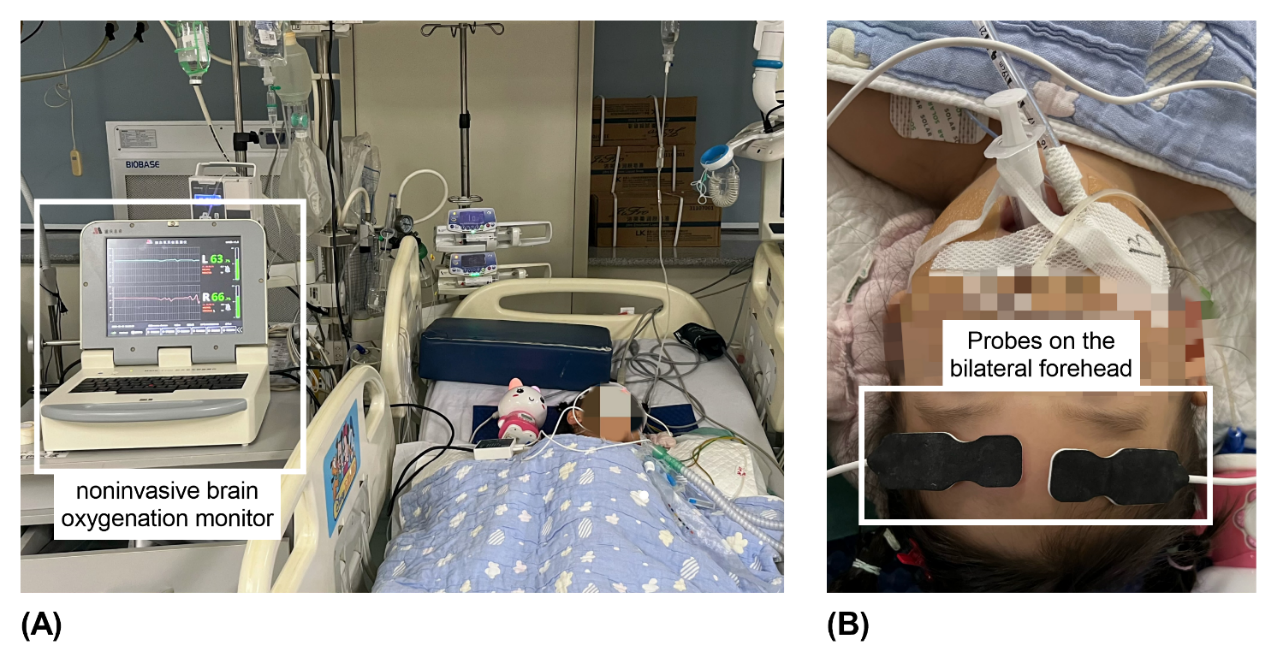

Supplement: Supplementary file 1 [file Table_1.DOCX]
